# How to evaluate the quality of the clinical learning environment in health professions education? Protocol of a systematic review

**DOI:** 10.1371/journal.pone.0293773

**Published:** 2025-04-04

**Authors:** Matthias M. Walter, Slavko Rogan, Alexander P. Schurz, Evert Zinzen

**Affiliations:** 1 Science and Research, Physio Insight, Haslach im Kinzigtal, Baden-Württemberg, Germany; 2 Faculty of Physical Education and Physiotherapy, Department Movement and Nutrition for Health and Performance, Vrije Universiteit Brussel, Brussels, Belgium; 3 Department of Health Professions, Bern University of Applied Sciences, Bern, Switzerland; Iran University of Medical Sciences, IRAN, ISLAMIC REPUBLIC OF

## Abstract

**Background:**

Internships can constitute up to one third of the curriculum and during these internships, the foundation for developing specific health professional competencies is formed. The clinical learning environment (CLE) is a critical determinant of the overall quality of internships in health profession education, shaping students’ professional competencies and experiences.

**Objective:**

This systematic review aims to identify and categorize assessment tools available for evaluating the quality of the CLE in health professions education.

**Methods:**

This in the International Database of Education Systematic Reviews preregistered systematic review [IDESR000098] will consider peer-reviewed articles in English where instruments are developed and validated to illustrate the quality of the CLE in higher education health professions students. The search strategy will encompass multiple electronic databases, including MEDLINE, EMBASE, the Cochrane Library, ERIC, Education Research Complete, Education Database, and CINAHL. Studies will be independently assessed for risk of bias using the COSMIN Risk of Bias checklist for systematic reviews of PROMs. We will summarize and tabulate the basic characteristics of each identified tool and via a comprehensive table we will summarize the reported psychometric properties.

**Discussion:**

This systematic review protocol will outline a comprehensive approach to identifying and evaluating assessment tools for measuring the quality of the CLE in health profession students. It is assumed that the findings will offer several notable advantages and impacts, which could significantly influence the quality of clinical education for health profession students.

## Introduction

Clinical education (CE) is a pivotal component of health professional education. It can be divided into two main parts: first, practical education in courses at the university and second, in internships where students are exposed to genuine clinical practice. Internships could constitute up to one third of the curriculum [1,2] and during these internships, the foundation for developing specific health professional competencies is formed [2]. Given its substantial role in shaping future practitioners, maintaining high-quality experiences in internships is of paramount importance to ensure the competence and excellence of future health professionals [3]. The key component to ensure the necessary competence is the clinical learning environment (CLE). It provides the real-life context for future health professionals, to observe role models and practice skills [4]. Furthermore, the CLE covers a complex social context for students in which they practice their skills while maintaining patient safety [5]. Moreover, learning outcomes of students are influenced by their perceptions of the CLE and a positive CLE is fundamental to the quality of students’ clinical experience [6] and irreplaceable in preparing students for their future health professional role [5,7,8]. However, factors such as human resources, materials, and other elements that can influence the CLE are primarily managed by clinical placements sites and therefore beyond the control of Directors of CE at higher educational institutions. Nevertheless, it is the responsibility of Directors of CE to provide high-quality CE experiences [9] and learning outcomes are increasingly recognized as being shaped by the quality of the CLE within clinical placements [10]. Assessing the CLE is therefore essential to ensure the competence and excellence of future health professionals.

A previous review cataloged assessment tools across multiple health professions, however, its methodology did not fully encompass all relevant aspects [11]. For instance, the narrow inclusion of specific keywords, such as “physical therapy,” may have resulted in the omission of tools particularly pertinent to physiotherapy education. To the best of our knowledge, no tools have been specifically developed to assess the CLE within physiotherapy education. Nevertheless, the 21 instruments identified in this review, originally created for other health professions, provide valuable insights into CLE assessment across various disciplines. These instruments not only facilitate the exploration of shared best practices but also underscore significant gaps, such as the lack of tools tailored to specific fields like physiotherapy. Including multiple health professions in this research offers a comprehensive perspective on the value of the CLE across various disciplines. While the specific contexts and requirements differ among professions, the foundational elements of high-quality CLE—such as effective supervision, resource availability, and interprofessional collaboration—are consistent.

The goal of this systematic review is to identify and catalog available tools for assessing the CLE in health professions education. By presenting a comprehensive analysis, the review aims to uncover gaps and highlight opportunities to refine the measurement and enhancement of CLE quality across diverse health disciplines.

## Methods

This systematic review has been preregistered in the International Database of Education Systematic Reviews under the registration number [IDESR000098] and follows the guidelines of the Consensus‐based Standards for the selection of health Measurement Instruments (COSMIN) methodology for conducting systematic reviews of Patient‐Reported Outcome Measures (PROMs) [12–14] and the Preferred Reporting Items for Systematic reviews and Meta-Analyses (PRISMA) for reporting the research process [15]. Any significant updates or changes to this protocol will be documented in the same registration entry to ensure transparency and traceability throughout the research process. To ensure transparency, the complete search strategies for all databases, as well as a detailed list of studies excluded during the full-text screening phase with specific reasons for exclusion, will be included in the final report.

### Ethics and dissemination

Due to the nature of a systematic review and its secondary data analysis ethical review is not required. The results of this systematic review will be disseminated through a peer-reviewed publication to ensure the rigor and credibility of the findings.

### Research question

This systematic review aims to answer the following research question: Which assessment tools are currently available to evaluate the quality of the CLE in health profession education?

### Eligibility criteria

This systematic review considers peer-reviewed articles in English language in which self-reported questionnaires completed by students are developed and validated (testing, evaluating) to assess the quality of the CLE in higher education health professions. We will define no time limit to the publication date. Articles focusing on other aspects of CE (e.g., postgraduate internships such as medical residency programs), tools targeting clinical supervisors’ evaluations, study protocols, abstracts, conference papers, and dissertations will be excluded. We will exclude grey literature to ensure methodological rigor and to focus exclusively on peer-reviewed studies, which are typically subject to a higher standard of quality assurance.

### Information source

The search strategy will encompass multiple electronic databases, including MEDLINE, EMBASE (accessed via Ovid), the Cochrane Library, ERIC, Education Research Complete, Education Database and CINAHL (accessed via Ebsco).

### Search strategy

We will use the SPIDER (Sample/ Phenomenon of Interest/ Design/ Evaluation/ Research Type) framework which is recommended for mixed methods qualitative research [16]. A comprehensive search strategy will be conducted to identify relevant studies. The search strategies will encompass key words as well as Medical Subject Headings (MeSH terms) depending on the database and will be supplemented by synonyms. We decided not to include specific research types (e.g., “Mixed methods,” “Qualitative,” “Quantitative”) in our search strategy. This decision was made to ensure that potentially relevant articles focusing on the assessment tools and their properties are not excluded solely based on methodological labels.

A pilot search will focus on the electronic database MEDLINE (PubMed). The researchers will analyze text words in article titles, abstracts, and index terms related to the research topic. The identified keywords and index terms will be customized for each information source and combined using Boolean operators, forming the final search strategy. In addition to electronic databases, the researchers will explore the reference lists of included studies to uncover further relevant studies. The process of identifying relevant studies will be completed by the end of March 2025. Table 1 represents the pilot search strategy.

**Table 1:** Search strategy.

Search strategy: Sample/ Phenomenon of Interest/ Design/ Evaluation/ Research Type	
Sample	- Physiotherapy - Physical therapy - Nurse - Nursing - Medical students - Allied health professionals - Occupational therapy - Occupational therapists - Speech therapy - Speech-language pathology - Speech therapists - Dietetics - Dietitians - Pharmacists - Pharmacy students - Dental students - Dentistry - Respiratory therapy - Respiratory therapists - Medical technology - Medical technologists - Health profession students - Healthcare students - Physical therapy - Nurse - Nursing - Medical students - Allied health professionals - Occupational therapy - Occupational therapists
Phenomenon of Interest	- Internships - Clinical placement - Clinical education - Clinical training - Practicum - Fieldwork - Clinical rotation - Clinical experience - Professional practice - Clinical practicum - Work-based learning - Workplace learning - Clinical supervision - Clinical teaching - Experiential learning - Hands-on training - Apprenticeship AND - Quality - Clinical learning environment
Design	- Observational - Cohort - Survey - Focus group - Interview - Validation - Reliability
Evaluation	- Instrument - Questionnaire - Tool - Survey - Scale - Inventory - Evaluation - Assessment - Measurement
Pilot search string for PubMed	(((((“Physiotherapy” OR “Physical therapy” OR “Physiotherapist” OR “Physical therapist” OR “Nurse” OR “Nursing” OR “Nursing students” OR “Medical students” OR “Allied health professionals” OR “Occupational therapy” OR “Occupational therapist” OR “Speech therapy” OR “Speech-language pathology” OR “Speech therapist” OR “Dietetics” OR “Dietitian” OR “Pharmacy” OR “Pharmacy students” OR “Pharmacist” OR “Dental students” OR “Dentistry” OR “Respiratory”)) AND (((“Internships” OR “Clinical placement” OR “Clinical education” OR “Practicum” OR “Fieldwork” OR “Work placement” OR “Clinical rotation” OR “Clinical training” OR “Professional practice” OR “Clinical experience” OR “Residency” OR “Apprenticeship” OR “Hands-on training” OR “Clinical attachment” OR “Practical training”)) AND ((Clinical learning environment) OR (quality)))) AND ((“Observational” OR “Cohort” OR “Survey” OR “Focus group” OR “Interview” OR “Validation” OR “Reliability” OR “Case study” OR “Cross-sectional” OR “Longitudinal” OR “Qualitative” OR “Quantitative”))) AND ((“Instrument” OR “Questionnaire” OR “Tool” OR “Survey” OR “Scale” OR “Inventory” OR “Evaluation” OR “Assessment” OR “Measurement”))) 3,185 results

### Selection of sources of evidence

First, two reviewers (MW, AS) will independently screen title and abstract for eligibility criteria. Any articles deemed relevant by both reviewers will be included in the full-text review. If the relevance of a study was unclear from the abstract, then the full article will be ordered. Second, two investigators (MW, AS) will conduct an independent assessment of the full-text articles to ascertain their compliance with the predefined eligibility criteria. To gauge the inter-rater agreement, Cohen’s κ statistic will be computed for both the initial title and abstract review stage, as well as for the subsequent full article review stage. Cohen’s κ values will be interpreted as follows: < 0.20 indicates poor agreement, 0.21–0.40 fair agreement, 0.41–0.60 moderate agreement, 0.61–0.80 substantial agreement, and > 0.81 almost perfect agreement [17]. This will ensure consistent and transparent reporting of inter-rater reliability. In cases where any discrepancies arise during the evaluation of full-text articles, a reevaluation of these discordant articles will be performed. Any persisting disagreements regarding the eligibility of studies will be diligently resolved through constructive discussions with a third reviewer (SR). The goal of these discussions will be to achieve complete consensus among all reviewers regarding the inclusion of studies. We will use Rayyan for the screening process [18].

### Data collection process and risk of bias assessment

Data from the articles included will be extracted independently by two team members (MW, AS) into a data extraction table. The extracted data will include details about the authors, health profession investigated, purpose of the instrument, instrument design, availability, scoring method, validation-study sample characteristics, as well as psychometric properties such as reliability and validity of the instrument. Additional information will include the location(s) of data collection, other relevant sociodemographic information, publication year, study design, study methods, results, and authors’ interpretation or conclusion. To identify any necessary adjustments to the data extraction table, we will conduct a pilot test using three full texts before proceeding with the full data extraction. In case of any disagreements, a third reviewer (SR) will be involved to reach consensus. Where required, authors will be contacted to request missing or additional data. Studies will be assessed with respect to risk of bias independently by two reviewers (MW, AS) using the COSMIN Risk of Bias checklist for systematic reviews of PROMs [12]. A consensus meeting will be organized with a third researcher (SR) in case of disagreement. The data will be entered into an Excel spreadsheet developed according to COSMIN recommendations. Initially, a test run with three studies will be conducted, and the spreadsheet will be adapted based on reviewer feedback.

### Effect measures

The primary aim of this systematic review is to identify and list the assessment tools currently available for measuring CLE in health profession education. While the main focus is on cataloging the tools, we will also report on key measurement properties to provide context regarding their reliability and validity. We will categorize the measurement properties as follows:

Reliability: Evaluated using measures such as Cronbach’s alpha for internal consistency and Intraclass Correlation Coefficient (ICC) or weighted kappa for test-retest reliability. Tools with reported reliability coefficients of ≥ 0.70 will be considered to have sufficient reliability.

Validity: Assessed through content validity (expert evaluations), construct validity (correlations with similar instruments), and criterion validity (comparison with gold standards or external criteria). A correlation coefficient of ≥ 0.70 will be considered indicative of sufficient validity.

Measurement Error: Evaluated using metrics such as Standard Error of Measurement (SEM) and Minimal Detectable Change (MDC), with thresholds based on minimal important change.

Responsiveness: Measured by the tool’s ability to detect change over time, with an area under the curve (AUC) value of ≥ 0.70 considered sufficient.

Interpretability: Assessed using Minimal Important Change (MIC) and Minimal Important Difference (MID), where available.

Each measurement property will be summarized to provide a comprehensive overview of the tool’s effectiveness in measuring the quality of internships. The findings will be categorized as “sufficient,” “insufficient,” or “indeterminate” based on the reported data and predefined thresholds [13].

### Synthesis of results

We will summarize and tabulate the basic characteristics of each identified tool, including the name, authors, publication year, health profession targeted, purpose, and design of the tool. Details regarding the target population, setting of the internships, and administration methods will also be included. Additionally, a comprehensive table will be created to summarize the reported psychometric properties for each tool. This table will include columns for reliability metrics (e.g., Cronbach’s alpha, ICC), validity metrics (e.g., content validity, construct validity), measurement error (e.g., SEM, MDC), responsiveness, and interpretability. Table 2 shows an example.

**Table 2:** Example of Data Synthesis.

Tool Name	Authors	Year	Health Profession	Purpose	Design	Reliability	Validity	Measurement Error	Responsiveness	Interpretability
Tool A	Smith et al.	2020	Nursing	Assess internship quality	Survey	Cronbach’s alpha: 0.85	Construct validity: good	SEM: low	AUC: 0.75	MID: 5
Tool B	Jones et al.	2018	Physiotherapy	Evaluate clinical placements	Questionnaire	ICC: 0.78	Content validity: adequate	MDC: moderate	AUC: 0.80	MIC: 4

The results of the risk of bias assessment, conducted using the COSMIN Risk of Bias checklist, will be summarized for each tool. A separate table will be created to present the risk of bias for each study. Table 3 shows an example.

**Table 3:** Example of Risk of Bias Assessment.

Tool Name	Authors	Year	Health Profession	Overall Risk of Bias	Reliability RoB	Validity RoB	Measurement Error RoB	Responsiveness RoB	Interpretability RoB
**Tool A**	Smith et al.	2020	Nursing	Low	Low	Low	Low	Low	Low
**Tool B**	Jones et al.	2018	Physiotherapy	Moderate	Low	Moderate	High	Moderate	Low

Furthermore, we will group the tools by health profession, allowing us to identify areas with numerous tools and those with few or no tools. This grouping will help determine the availability and distribution of tools across different health professions.

### Reporting bias assessment

To assess the potential for reporting bias in the included studies, we will implement several strategies. First, we will search for study protocols and trial registrations to identify any discrepancies between the planned and reported outcomes. Additionally, we will compare the reported findings with the original research questions and objectives stated in the methods sections of the included studies. If necessary, authors will be contacted to clarify any inconsistencies. For each study, we will note whether all prespecified outcomes were reported and whether results were selectively reported based on statistical significance.

### Certainty assessment

If possible, the certainty of evidence will be evaluated using the GRADE approach (Grading of Recommendations Assessment, Development and Evaluation) as recommended by the COSMIN guidelines for conducting systematic reviews of PROMs [13]. This involves assessing the quality of evidence across the following domains: risk of bias, consistency of results, directness of evidence, precision of estimates, and publication bias. Each domain will be rated as high, moderate, low, or very low. The overall certainty of evidence for each outcome will be determined based on these assessments. Two reviewers (MW, AS) will independently evaluate the certainty of evidence, and any discrepancies will be resolved through discussion with a third reviewer (SR). The results of the certainty assessment will be presented in summary of findings tables, which will provide a clear overview of the quality of evidence supporting the main findings of the review.

## Limitation

One notable limitation of this systematic review protocol is the potential for publication bias. Since the study relies primarily on published works, there is a possibility that relevant research remains inaccessible due to publication restrictions or database coverage. This could lead to an incomplete representation of the available evidence.

Another limitation is the variability in scientific backgrounds and needs across different health professions. The instruments designed for one health profession might not be directly applicable or relevant to another, which could complicate comparisons and synthesis. To address this, we will conduct subgroup analyses based on health profession to provide more tailored insights and ensure the relevance of our findings to each specific field.

Additionally, the use of the SPIDER framework, which is designed for mixed methods qualitative research, may introduce bias by being too specific. This specificity might limit the scope of included studies, potentially excluding relevant quantitative research that could contribute valuable data. To counter this, we will employ a flexible approach in our search strategy, adapting and expanding our keywords and inclusion criteria as necessary to capture a broader range of studies.

## Discussion

This systematic review protocol outlines a comprehensive approach to identifying and evaluating assessment tools for measuring the quality of internships during clinical education in health profession students. The findings of this systematic review offer several notable advantages and impacts, which could significantly influence the quality of clinical education for health profession students. One major benefit is the potential to standardize the assessment of internship quality. By identifying and evaluating reliable and validated assessment tools, this review can help ensure that all institutions use consistent and comparable methods for evaluating internships. This standardization is crucial for maintaining high educational standards across different programs and institutions.

The application of robust assessment instruments can lead to better monitoring and improvement of internship programs. Educational institutions will be able to identify areas needing improvement and implement changes that enhance the overall quality of education and the learning experiences of students.

Furthermore, the results of this review will provide educational institutions with the evidence needed to make informed decisions regarding the selection and implementation of assessment tools. Institutions can choose the tools that best meet their specific needs, ensuring that the evaluations are both relevant and effective.

Finally, identifying gaps and weaknesses in existing assessment tools could stimulate further research and development. This could lead to the development of new or improved assessment instruments that are better tailored to the diverse needs of different health professions.

In summary, the results of this systematic review have the potential to bring about significant improvements in the quality and effectiveness of clinical education for health profession students. By providing an evidence-based foundation, this review supports the continuous enhancement and adaptation of assessment tools and methods to meet the evolving demands of healthcare education.

## Supporting information

S1 Checklist PRISMA 2020 checklist used to ensure the transparent and complete reporting of our systematic review. (DOCX)
